# Operative planning aid for optimal endoscopic third ventriculostomy entry points in pediatric cases

**DOI:** 10.1007/s00381-016-3320-y

**Published:** 2017-01-18

**Authors:** Zsolt Zador, David J. Coope, Abteen Mostofi, Ian D. Kamaly-Asl

**Affiliations:** 1 Department of Pediatric Neurosurgery, Royal Manchester Children’s Hospital, Manchester, M13 9WL UK; 2 Department of Neurosurgery, Greater Manchester Neuroscience Centre, Salford Royal NHS Foundation Trust, Salford, UK; 3 Institute of Cardiovascular Sciences, Centre for Vascular and Stroke Research, University of Manchester, Manchester, UK; 4 Wolfson Molecular Imaging Centre, The University of Manchester, Manchester, UK

**Keywords:** Endoscopic third ventriculostomy, Optimal ETV trajectory, Pediatric hydrocephalus

## Abstract

**Object:**

Endoscopic third ventriculostomy (ETV) uses anatomical spaces of the ventricular system to reach the third ventricle floor and create an alternative pathway for cerebrospinal fluid flow. Optimal ETV trajectories have been previously proposed in the literature, designed to grant access to the third ventricle floor without a displacement of eloquent periventricular structures. However, in hydrocephalus, there is a significant variability to the configuration of the ventricular system, implying that the optimal ETV trajectory and cranial entry point needs to be planned on a case-by-case basis. In the current study, we created a mathematical model, which tailors the optimal ETV entry point to the individual case by incorporating the ventricle dimensions.

**Methods:**

We retrospectively reviewed the imaging of 30 consecutive pediatric patients with varying degrees of ventriculomegaly. Three dimensional radioanatomical models were created using preoperative MRI scans to simulate the optimal ETV trajectory and entry point for each case. The surface location of cranial entry points for individual ETV trajectories was recorded as Cartesian coordinates centered at Bregma. The distance from the Bregma in the coronal plane represented as “x”, and the distance from the coronal suture in the sagittal plane represented as “y”. The correlation between the ventricle dimensions and the x, y coordinates were tested using linear regression models.

**Results:**

The distance of the optimal ETV entry point from the Bregma in the coronal plane (“x”) and from the coronal suture in the sagittal plane (“y”) correlated well with the frontal horn ratio (FHR). The coordinates for x and y were fitted along the following linear equations: x = 85.8 FHR−13.3 (*r* ^2^ = 0.84, *p* < 0.001) and y = −69.6 FHR + 16.7 (*r* ^2^ = 0.83, *p* < 0.001).

**Conclusion:**

The surface location of the optimal cranial ETV entry point correlates well with the ventricle size. We provide the first model that can be used as a surgical planning aid for a case specific ETV entry site with the incorporation of the ventricle size.

## Introduction

**Fig. 1:**
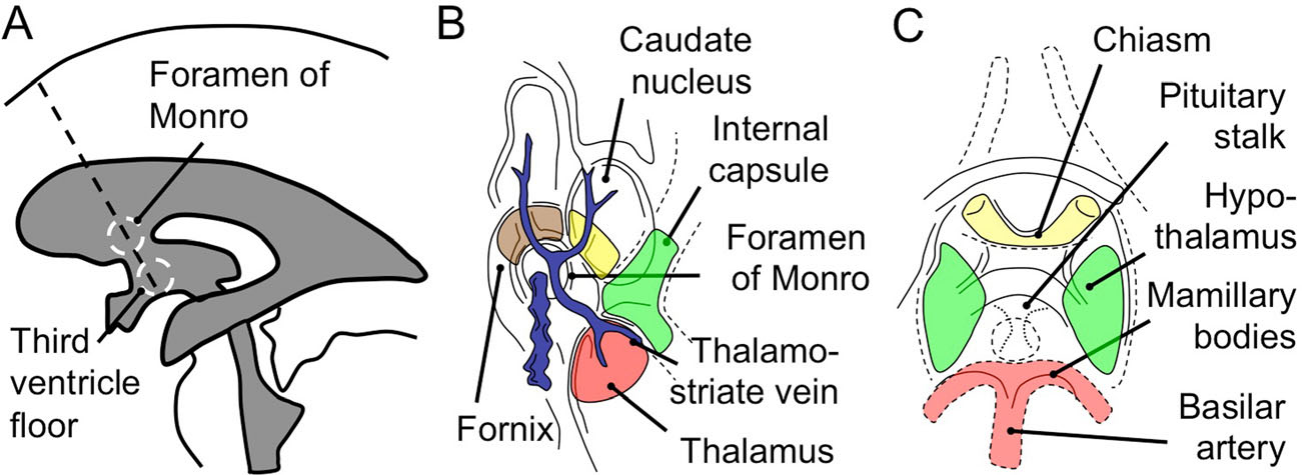
Surgical anatomy of the third ventriculostomy and the concept of optimal ETV trajectory. *A*: schematic showing optimal ETV trajectory (*interrupted line*) aligned along the foramen of Monro and the third ventricle floor. *B* and *C*: diagram depicting the operative view and eloquent periventricular structures at the level of the foramen of Monro (*B*) and the third ventricle floor (*C*). Panel B: eloquent structures are highlighted as follows, *brown*: fornix; *yellow*: caudate nucleus; *green*: genu of internal capsule; *red*: thalamus. Panel C: *yellow*: chiasm; *red*: basilar artery termination; *green*: hypothalamus. *Interrupted circle*: fenestration site

Endoscopic third ventriculostomy (ETV) is a minimally invasive procedure of cerebrospinal fluid (CSF) diversion widely used for treating hydrocephalus of various etiologies [1–3] with a success rate of 68.5–83% [1, 4–6]. This technique involves passing a rigid endoscope through the CSF spaces of the ventricular system to access and fenestrate the floor of the third ventricle. The linear working trajectory for this procedure passes adjacent to multiple eloquent periventricular structures such as the fornix, caudate nucleus, genu of the internal capsule, and hypothalamus (Fig. 1). Excessive manipulation to these structures can cause disturbance in motor, memory, speech, and endocrine functions leading to a permanent morbidity or mortality [7]. Furthermore, injury to adjacent vascular structures such as the choroid plexus, thalamostriate vein, or potentially major arteries can lead to devastating bleeding. Previous studies recommended optimal ETV entry points that provide an atraumatic trajectory avoiding these structures [8–10]. The location of these points however varies considerably which is paralleled by the highly diverse morphology of the ventricles in hydrocephalus [9, 11, 12]. These observations suggest that ETV entry points should be planned on a case-by-case basis with adjustments based on the ventricle sizes [13, 14]. Neuronavigation is now well-accepted in neurosurgical practice, however, it has not been widely used for ETV planning as most studies report using a single conventional transcortical entry point [1, 4–6, 14]. Furthermore, the infrastructure to this adjunct is not always available to the surgeon. We therefore examined the correlation between ventricular morphometry and the location of the optimal ETV entry points in 30 pediatric patients with a specific view to creating a surgical planning aid.

## Methods

Our study was part of a registered audit at the Clinical Audit Department of Royal Manchester Children’s Hospital (reference number 5028^14^). Our anonymized database contained 30 consecutive pediatric patients aged 6 months–16 years with varying degrees of ventriculomegaly who underwent treatment for newly diagnosed hydrocephalus (as previously reported [14]). We excluded cases with supratentorial mass lesions. We reconstructed preoperative MRI scans and analyzed them using a Brainlab iPlan workstation (software version 3.0). This technique allowed the creation of the optimal ETV trajectory in three dimensions. The “probe view” function allowed us to establish a view along the trajectory and visualize its relations relative to adjacent structures.

### Assessment of ventriculomegaly

**Fig. 2:**
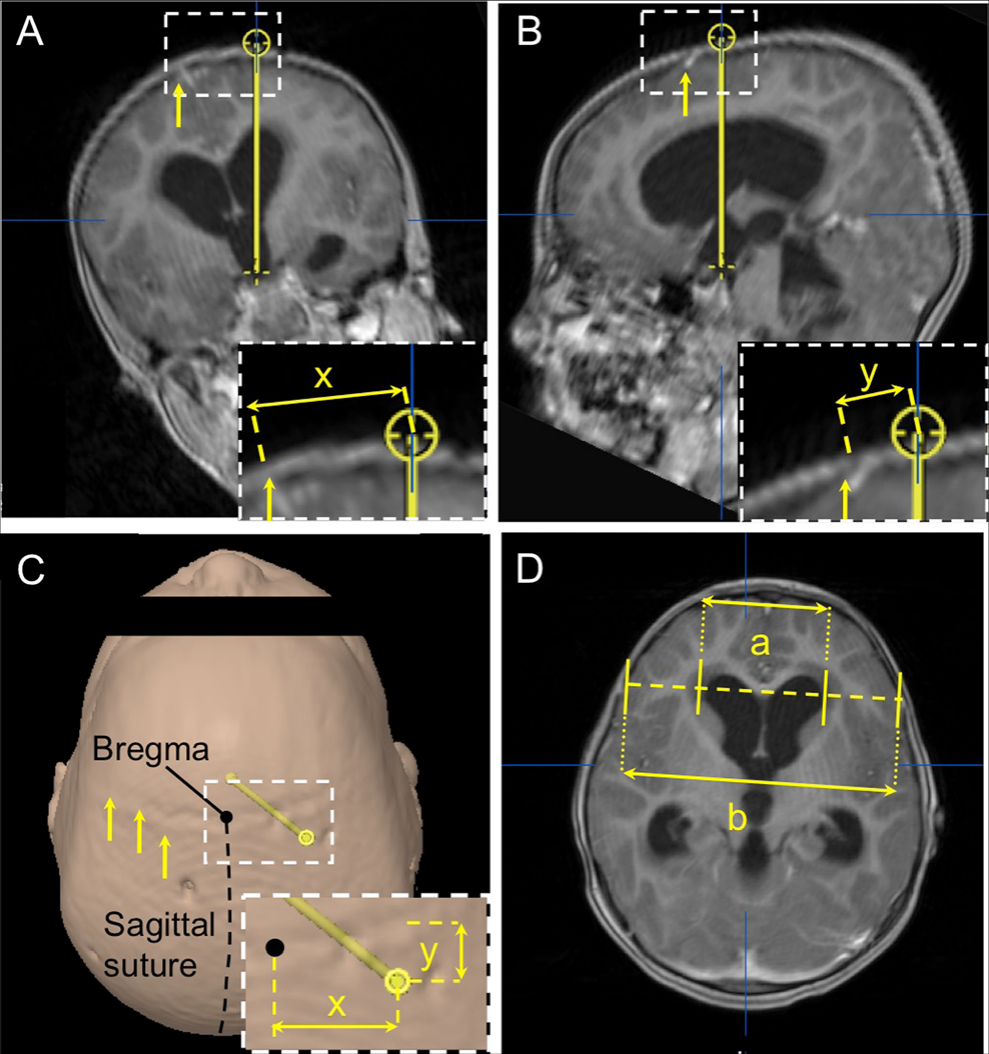
Radioanatomical analysis of optimal ETV trajectories. Panel A: coronal view of the optimal ETV trajectory (*yellow line*) connecting the foramen of Monro and the floor of the third ventricle. *Arrow* indicates the midline. Insert: measurement of ETV entry point (*yellow target*) distance from Bregma in the coronal plane (“x”). Panel B: sagittal view of the optimal ETV trajectory depicted in “A”. Arrows indicate the coronal suture. Insert: distance of the ETV entry point (“y”) form the coronal suture (*yellow arrow*). Panel C: surgical view of a three-dimensional model created for the case shown in A and B. Optimal ETV trajectory (*yellow bar*) and entry point (*yellow dot*) correspond to case in “A” and “B”. *Arrows* mark the coronal suture. Inset: “x” and “y” represent entry point distance from the Bregma and coronal suture, respectively. Panel D: frontal horn ratio expressed as the distance between the frontal horns (“a”) and the internal diameter of the skull along the same line (“b”)

The diversity of ventricular dimensions was quantified using the frontal horn ratio (FHR) as described by Hahn and Rim (1976) [15]. This parameter was calculated as the distance between the anterior angles of the two frontal horns (bifrontal distance) divided by the transverse internal diameter of the skull measured along the same line used to assess the bifrontal distance (Fig. 2d).

### Assessment of optimal ETV entry points and trajectory

The optimal trajectory for a right-sided ETV was defined as the line connecting the floor of the third ventricle with the center of the foramen of Monro [8, 10, 16, 17] (Fig. 2a and b). The optimal ETV entry point was derived as the intersection of this trajectory with the level of the skin. The location of this entry point was recorded as the distance laterally from Bregma in the coronal plane perpendicular to the sagittal suture (x) and the distance anteroposteriorly from the coronal suture in the sagittal plane parallel to the sagittal suture (y) (Fig. 2a and b). Entry points posterior to the coronal suture were expressed as negative “y” values.

### Data analysis

The correlation of the optimal entry points (x, y coordinates) was analyzed in relation to the frontal horn ratio using the curve fitting application in Microsoft Excel and R statistics (version 3.1.2, Foundation for Statistical Computing).

## Results

### Patients

The male:female ratio was 1.7:1 (19 males and 11 females) with an average age of 7.66 ± 4.59 (mean ± standard deviation). With regards to underlying neurosurgical diagnosis, posterior fossa neoplasm (22/30 patients) was the most common (including medulloblastoma, ependymoma, and meningioma), followed by primary CSF circulation abnormalities (8/30 patients). Mean frontal horn ratio was 0.38 and ranged between 0.24–0.5 (as previously reported [14]).

### Assessment of optimal entry point

**Fig. 3:**
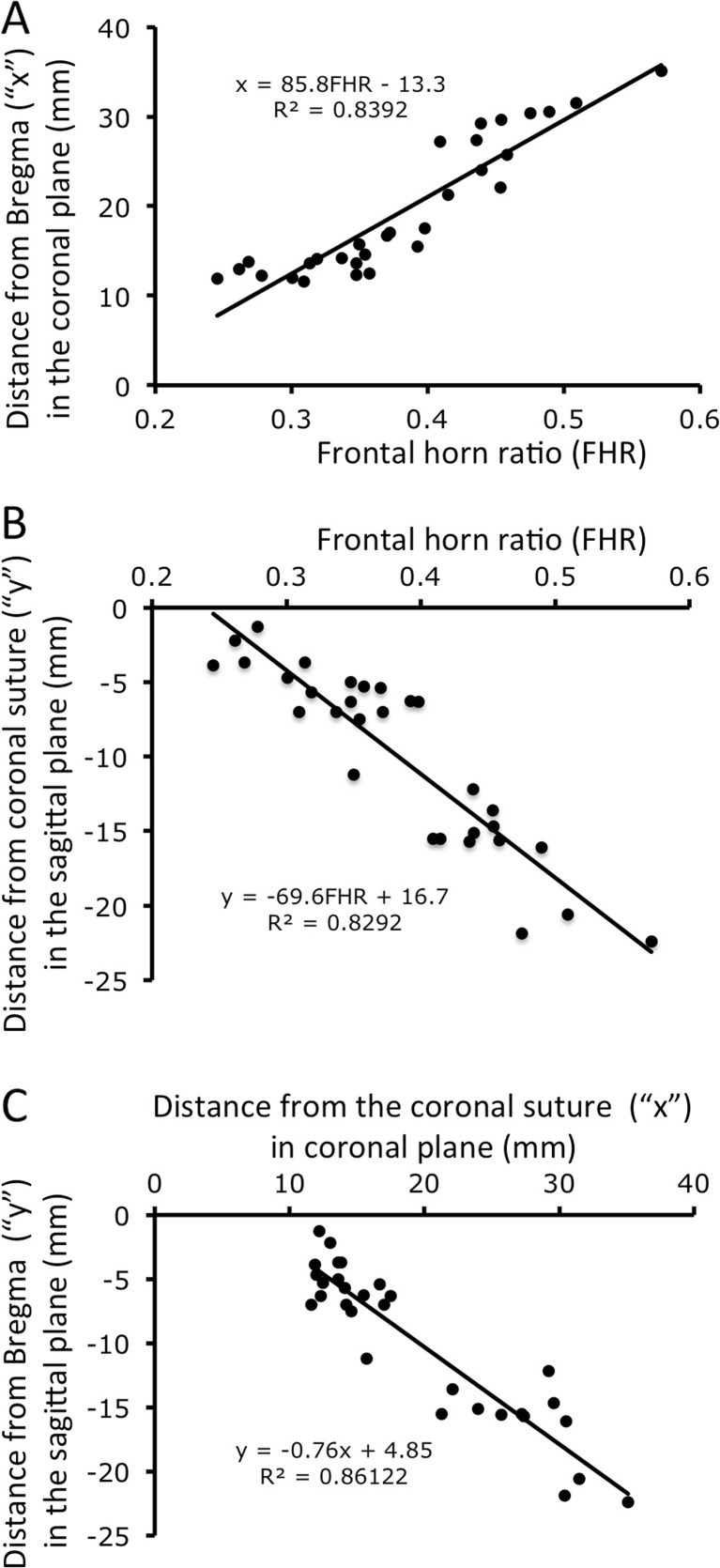
Optimal entry points are plotted in a Cartesian coordinate system with analysis of linear regression. Scatter plot of optimal entry point distances from Bregma in the midline along the coronal plane (*A*) and from the coronal suture in the sagittal plane (*B*) plotted against the frontal horn ratio (FHR). Note the linear correlation between the frontal horn ratio and both “x” and “y” variables. Panel C: labeling in the formulas follow Cartesian coordinate system reconstituting the skull surface with “0” indicating Bregma, “x” and “y” axis representing distances in the coronal and sagittal plane, respectively. Note the linear distribution of optimal entry points in a posterolateral direction with an increasing ventricle size. Linear equations with *r* ^2^ values are inserted in each graph; variable notations follow those in Fig. 2

The third ventricle floor was accessible in all cases therefore establishing the optimal ETV trajectory was feasible in every patient. The average distance of the optimal entry point from Bregma were 29.2 ± 9.4 mm (mean ± standard deviation, range 17.3 to 47.2 mm) in the coronal plane (“x” coordinate) and −12.8 ± 5.6 mm (range: −7.4 to −24.5 mm) posterior to the coronal suture in the sagittal plane (“y” coordinate) which is comparable to the values found by Duffner [9] and Cheng [8]. Numeric coordinate values of the optimal entry point showed a good correlation with the FHR: with x = 85.8 FHR−13.3 (*r* ^2^ = 0.84, *p* < 0.001) and y = −69.6 FHR + 16.7 (*r* ^2^ = 0.83, *p* < 0.001). Further analysis using multivariate regression showed that with an increasing ventricle size, the optimal entry point seemed to migrate more lateral and posterior relative to Bregma (*p* < 0.05 for both x, y variables). Scatter plot of the optimal entry points (Fig. 3c) fitted well to a linear model: y = −0.76× + 4.85 (*r* ^2^ = 0.86, *p* < 0.001).

## Discussion

The success and safety of an ETV relies upon an endoscope trajectory that grants good visualization and satisfactory access to the surgical target the third ventricular floor. This optimal trajectory runs between the tuber cinereum and the center of the foramen of Monro, and its extension to and intersection with the cranial surface marks the site of the optimal ETV entry point [8–10, 16, 17]. We analyzed the distribution of optimal ETV trajectories and correlated their pattern with the target ventricle size (expressed as the frontal horn ratio of Hahn and Rim). Our results demonstrate a good correlation between the craniometric location of the optimal ETV entry point and the target ventricle size. Analysis of the data trend shows that the entry site migrates more posteriorly and laterally with an increasing ventricle size, which can be predicted using a simple mathematical formula.

### Radioanatomical simulations of surgical approaches

The use of radioanatomical information in surgical simulations and preoperative planning are well-documented in the literature. The translation value in assessing individual relations of the asterion and transverse/sigmoid sinus junction in retrosigmoid craniotomies was demonstrated by multiple studies [18–20]. Our previous analysis of neuronavigation data from surgical phantoms [21] and operative cases [22] for surgical corridor size in the eyebrow craniotomy showed a good correlation between the simulations and intraoperative results. Radioanatomical data has been used to compare variants of the transpetrosal approach confirming the expected increments of working areas and angle with progressive bone removal [23]. The extent of tissue displacement in ETVs has been quantified by several investigators using intraoperative neuronavigation and paralleled with clinical safety of the procedure [14, 16, 17, 24]. Furthermore, several studies advocate surgical simulation that uses virtual platforms or physical heads as adjunct models of surgical training as well as preoperative planning [25–27]. Given above, there is an emerging methodological trend towards using radioanatomical simulations to incorporate individual anatomical characteristics in surgical planning as well as training.

### Optimal ETV trajectory

The concept of an atraumatic ETV entry point (i.e., optimal ETV entry point) was introduced by Kanner et al. [10], who suggested it to be 3 cm off the midline along the coronal suture and 1 cm anterior to it. This concept was also analyzed by Duffner [9] and Chen [8], who found the optimal entry point was more posterior (1 cm behind the coronal suture) within the same sagittal plane. Each of these studies found a relatively high variability for the craniometric location of these entry points with an overall range of 12.5 to 44.4 mm for the distance to the midline and 30.6 mm anterior to 46.5 mm posterior relative to the coronal suture. We recently compared three frequently used ETV entry points (precoronal, coronal, and posterior-coronal points) in the same patient cohort with regards to their breach of eloquent periventricular structures in pediatric patients with varying degrees of ventriculomegaly [14]. The results showed that none of the entry points granted a trajectory that avoided breaching eloquent periventricular structures in every case when accessing to the third ventricular floor. Stratifying the entry points by the target ventricle size showed that a more posterior entry point was associated with a less frequency of tissue breach for larger ventricles. These results suggested that there is no single best ETV entry point that is applicable to all cases of ventriculomegaly. Instead, entry points are better planned on a case-by-case basis with the incorporation of ventricle size [13].

### ETV planning aid with the incorporation of ventricle size

Ventricle dimensions can vary significantly in hydrocephalus. Lateral ventricle height and interventricular distance ranged between 25.2–60.0 mm and 37.6–89.1 mm respectively in a study of 30 adult patients with hydrocephalus [9]. In a study of 140 adult patients with hydrocephalus related to subarachnoid hemorrhage [12], the largest axial cross-sectional area of the third ventricle ranged on a scale of up to sixfold. Such variance in the lateral and third ventricle dimensions will translate into different relations between the third ventricle floor and the foramen of Monro causing a shift in the locations of the optimal ETV entry point. Supporting this observation (as discussed above), a high variability has been noted for the location of the optimal ETV entry point by multiple studies [9, 10, 15–17]. Although neuronavigation allows preoperative planning and individualization of ETV approaches to match these variations, the infrastructure for this may not be available in all centers. There is also the element of associated financial cost and the time required to set up these systems in the emergency setting. Furthermore, in several case series, the authors quote a single entry point for ETVs [1–6] suggesting a generally accepted surgical practice not to use neuronavigation routinely. In search of an operative planning aid, we have created a mathematical formula that incorporates the target ventricle size using the FHR and computes the location of the optimal ETV entry point. With a single input parameter, our model allows a computation of ETV entry point distance from the midline and the coronal suture, which can be applied in preoperative planning. The proximity of critical anatomical structures in particular the superior sagittal sinus, primary motor cortex should additionally be taken into account.

## Conclusion

Our study demonstrates a strong correlation between the ventricle size and the optimal ETV entry point in pediatric patients. This has allowed the creation of an operative planning aid, which we are proposing to verify in operative cases.
